# 5’-adenosine monophosphate mediated cooling treatment enhances ΔF508-Cystic Fibrosis Transmembrane Conductance Regulator (CFTR) stability *in vivo*

**DOI:** 10.1186/s12929-015-0178-3

**Published:** 2015-09-04

**Authors:** Yueqiang Zhang, William G. O’Brien, Zhaoyang Zhao, Cheng Chi Lee

**Affiliations:** Department of Biochemistry and Molecular Biology, University of Texas Health Science Center at Houston, Houston, TX 77030 USA

**Keywords:** Misfolded protein, Mutant, Genetic disorder, Temperature-sensitive, Whole body cooling, Rescue, Treatment, Hypothermia, Hypometabolism, Cystic Fibrosis

## Abstract

**Background:**

Gene mutations that produce misprocessed proteins are linked to many human disorders. Interestingly, some misprocessed proteins retained their biological function when stabilized by low temperature treatment of cultured cells *in vitro*. Here we investigate whether low temperature treatment *in vivo* can rescue misfolded proteins by applying 5’-AMP mediated whole body cooling to a Cystic Fibrosis (CF) mouse model carrying a mutant cystic fibrosis transmembrane conductance regulator (CFTR) with a deletion of the phenylalanine residue in position 508 (ΔF508-CFTR). Low temperature treatment of cultured cells was previously shown to be able to alleviate the processing defect of ΔF508-CFTR, enhancing its plasma membrane localization and its function in mediating chloride ion transport.

**Results:**

Here, we report that whole body cooling enhanced the retention of ΔF508-CFTR in intestinal epithelial cells. Functional analysis based on β-adrenergic dependent salivary secretion and post-natal mortality rate revealed a moderate but significant improvement in treated compared with untreated CF mice.

**Conclusions:**

Our findings demonstrate that temperature sensitive processing of mutant proteins can be responsive to low temperature treatment *in vivo*.

## Background

Increasing evidence has indicated that incorrect folding of proteins is the underlying cause of many human disorders and diseases, including Cystic Fibrosis (CF), Amyotrophic lateral sclerosis (ALS), Parkinson’s disease (PD) and Alzheimer’s disease (AD) [1, 2]. In a healthy cellular environment, misfolded proteins fail to pass the “quality control” of the endoplasmic reticulum (ER) and are retained and selectively degraded without being exported. These are typically described as trafficking defects since the misfolded proteins fail to reach their target sites. Thus, these disorders result from the loss of functional protein, as in CF [3–5]. In other cases, such as Parkinson’s and Alzheimer’s disease, some of the misfolded proteins evade the “quality control” of the ER, and form aggregates that disrupt normal cellular processes [6].

In efforts to identify ways to correct protein misfolding, researchers have found that many of the misfolding defects are temperature-sensitive and can be corrected, or partially corrected, by low temperature treatment of cultured cells. For example, congenital long QT syndrome is caused by mutations in the human ether-a-go-go-related gene (HERG) encoding a potassium channel. When cells were incubated at low temperature (27 °C), the expression of two trafficking-deficient mutations of long QT, HERG N470D and HERG R752W, resulted in improved incorporation of functional channels into the plasma membrane [7]. The receptor tyrosine kinase (RET) plays a critical role in the development of the kidney and the enteric nervous system. Missense mutations prevent its expression at the cell surface and lead to Hirschsprung disease, but low temperature (30 °C) could largely rescue the trafficking defect of the mutant protein [8]. Similarly, the common H1069Q mutation responsible for Wilson’s disease is due to a defect in the trafficking of this copper-dependent protein which can be rescued by lowering the temperature to 28 °C [9]. Two mutations in the pancreatic ATP-sensitive potassium channels A116P and V187D, located in the SUR1 subunit, reduce channel activity leading to persistent infancy hyperinsulinemic hypoglycemia. The temperature sensitive misfolding of each mutated protein observed at 37 °C was completely rescued at 18 °C [10]. Tyrosinase is an enzyme present in melanocytes involved in the synthesis of melanin. Mutations in this enzyme that retain the protein in the endoplasmic reticulum can cause oculocutaneous albinism. The Golgi processing and transport of the tyrosinase mutants to melanosomes was promoted by reducing the incubation temperature from 37 to 32 °C [11].

The low temperature rescue of mutant proteins causing CF has been intensively studied. CF is caused by mutations in the gene encoding the cystic fibrosis transmembrane conductance regulator (CFTR). The CFTR is an anion channel critical for fluid and electrolyte homeostasis at the epithelial surfaces of many organs, including lung, salivary glands and intestine. The loss of functional CFTR channels at the plasma membrane disrupts ionic (Cl^−^, Na^+^, HCO3^−^) homeostasis, which leads to thickened mucus, obstructed secretion ducts and reduced hydration of organs. The most common mutation is a triple nucleotide deletion in the CFTR gene, resulting in deletion of a phenylalanine at amino acid position 508 of the CFTR protein (ΔF508). This mutation accounts for approximately 70 % of defective CFTR alleles [12] and 90 % of CF patients in the United States carry at least one such allele [13]. Studies have shown that the mRNA for ΔF508-CFTR is transcribed and translated, but the mutant protein remains trapped in the ER, where it is recognized as a misfolded protein and rapidly degraded by the ubiquitin proteasome pathway before it can reach the plasma membrane [14]. Early studies showed that some post-translational processing of ΔF508-CFTR is temperature sensitive [15, 16]. When expressed in cells at 37 °C, ΔF508-CFTR is present predominantly as a 130 kDa unglycosylated form (band A). When cells are grown at a lower temperature (26 °C), the percentages of the 150 kDa core-glycosylated form (band B) and the mature fully glycosylated form (170 kDa, band C) were each increased. The percentage of ΔF508-CFTR in complex glycosylation forms progressively increased as incubation time at the reduced temperature (26 °C) progressed. The rescued ΔF508 was delivered to the plasma membrane and exhibited significant Cl^−^ transport activity [16, 17]. Decreasing the temperature to 16 °C was shown to be more effective in stabilizing ΔF508-CFTR in cultured cells [18]. A recent study further showed that low temperature treatment facilitated the restoration of ΔF508-CFTR activity in dissected intestinal tissue maintained *in vitro* [19]. These studies raise the possibility that a reduction in whole body temperature may attenuate the processing defect of mutant CFTR and restore all or some function *in vivo*. However, it remains unclear whether ΔF508-CFTR, after cooling *in vivo,* would behave in a similar manner to that shown *in vitro,* as reducing whole body temperature for such a study has not been reported.

Previously, we have shown that 5’-AMP induces reversible hypometabolism in large and small mammals [20, 21]. 5’-AMP induced hypometabolism (AIHM) was used to reduce core body temperature (T_b_) of mice to about 16 °C for several hours at an ambient temperature (T_a_) of 15 °C while allowing the animal to safely recover to the euthermic state [22, 23].

The aim of the present study is to investigate whether a whole body cooling strategy can rescue the temperature-sensitive misfolding and processing defect of a mutant protein *in vivo*. We have chosen to investigate the efficacy of AIHM in rescuing ΔF508-CFTR and restoring its function in CF mice, *in vivo*.

## Methods

### Mice

Cftr^F508 del^ mice (B6.129S6-*Cftr*^*tm1Kth*^/J), a mouse model for CF caused by the ΔF508 mutation, are used in this study [24]. Heterozygote parent CF mice and wild type mice were purchased from the Jackson Laboratory. All mice used in this investigation were provided with regular chow and water ad libitum. All animals are monitored at least once every 24 h by laboratory personnel or animal care staff. Mice were housed in standard husbandry conditions under a 12-h/12-h light/dark cycle at an ambient temperature (Ta) of 23 °C. Although both homozygote CF males and females are fertile, the females appeared to have poor capability to rear their young. Thus, each cage started with one homozygote male mating with two heterozygous females, and the males were removed after 2 weeks. Each pregnant female typically delivered 6–8 pups. Cages were examined daily to monitor the approximate time of birth and to assess mortality. Tissue samples from deceased animals were collected for genotyping and tail clips from live animals were collected at weaning for genotyping using a PCR method provided by the Jackson Laboratory. The studies were carried out under animal protocols HSC-AWC-09-010 approved by the Animal Welfare Committee (AWC), which is the institutional animal care and use committee (IACUC) at the University of Texas Health Science Center - Houston. All care and use of animals for this study was in full compliance with animal welfare guidelines of the AWC.

### Induction of deep hypometabolism in CF mice

Adenosine 5’-monophosphate was obtained from Sigma (St Louis, MO, USA). A freshly prepared 5’-AMP solution in phosphate buffered saline was administered to mice by intraperitoneal injection (ip) at a dosage of 0.5 mg/g body weight. After 5’-AMP injection, mice were maintained at a T_a_ of 15 °C, in separate beakers to facilitate cooling. Once mice entered deep stupor with a T_b_ of 16–17 °C, they were transferred to microisolator cages with bedding kept at a T_a_ of 15 °C. After keeping mice in the stupor state for up to 10 h, T_a_ was increased in a gradient manner from 15 °C to 17 °C to enhance arousal and re-warming. For CF mice undergoing a longer period of stupor, the T_a_ was kept at 14.5 °C using a refrigerated Comprehensive Lab Animal Monitoring System (CLAMS, Columbus Instruments) and 200 μl of saline was administrated at 7 h after administration of 5’-AMP. Mice that remained in stupor after 17 h were gradually rewarmed to induce arousal. All re-warming took place in a cage with food and water ad libitum. Oxygen consumption and carbon dioxide production rates were also monitored. Mouse body temperature (T_b_) was manually monitored by a digital thermometer (Traceable, Fisher; catalogue no. 15-077-8) placed into the rectum [21].

### Immunofluorescence staining

Briefly, the duodenum, defined as the first 3 cm of the small intestine, was dissected from wild type, untreated CF and CF mice treated by whole body cooling. The duodenum was fixed with 4 % paraformaldehyde solution and then embedded in paraffin before being sectioned. Paraffin-embedded mouse small intestine was sectioned in 5 μm thick slices. For anti-CFTR staining, sections were pretreated by heat-induced epitope retrieval, blocked in 10 % goat serum for 1 h and then incubated overnight with primary antibodies for CFTR (NB100-92156, Novus Biologicals) at 1:100 in incubation medium (PBS with 3 % goat serum, 3 % BSA, and 0.3 % Triton × 100). After four 5 min washes in washing buffer, the sections were incubated with secondary antibodies (Alexa Fluor 555–labeled goat anti-rabbit IgG; Invitrogen) for 1 h at room temperature at a concentration of 2 μg/ml in incubation medium. After four washes for five min each, cover slips were mounted with SlowFade Gold antifade reagent with DAPI (Invitrogen). Some slides were co-stained with wheat germ agglutinin, Alexa Fluor® 488 conjugate (Invitrogen), to visualize mucin secreted by goblet cells in mouse intestine.

### Mucin staining

WT or CF mouse small intestines were fixed overnight in cold Carnoy’s solution (ethanol-acetic acid-chloroform at a ratio of 6:3:1 by volume). The fixed tissues were processed in paraffin and cross sections (5 μm thick) were prepared. The sections were stained for mucin using periodic acid-Schiff (PAS)-Alcian blue (AB; pH 2.5) [25, 26].

### Isolation of mouse intestinal epithelium

The duodenal epithelium from the crypt and villus regions was isolated by a modification of the Flint et al. (1991) method [27]. Briefly, the small intestine duodenum was removed, inverted, mounted on a 4 mm diameter glass rod and divided into 2–3 mm lengths. The inverted segments were flushed by constant stirring in 100 ml of cold Hanks’ solution containing 0.5 mM dithiothretol at 4 °C for five min, transferred to 150 ml of cold chelating buffer (0.5 mM dithiothretol, plus protease inhibitor cocktail) and incubated at 4 °C with constant stirring for 20 min. The chelating buffer (pH 7.3) included 27 mM trisodium citrate, 5 mM Na_2_HPO_4_, 96 mM NaCl, 8 mM KH_2_PO_4_, 1.5 mM KCl, 0.5 mM dithiothreitol, 55 mM D-sorbitol and 44 mM sucrose. The detached epithelium was collected by centrifugation at 3,000 x g at 4 °C for 8 min and the pellets were suspended in 5 ml lysis buffer containing 50 mM Tris–HCl (pH 7.4), 0.25 mM sodium deoxycholate, 150 mM sodium chloride, 2 mM EDTA, 0.1 mM sodium orthovanadate, 10 mM sodium fluoride, 1 mM PMSF, 1 % triton X-100, plus protease inhibitor cocktail (Roche), mixed vigorously on a vortex mixer, then kept on ice for 30 min and centrifuged at 14,000 rpm at 4 °C for 10 min. The supernatant was collected and stored at −80 °C [27].

### Western blot analysis

The protocol was largely as previously described [28]. Briefly, the detached epithelial cell lysates were mixed with 2X modified Laemmli Sample Buffer containing 8 M urea and incubated for 30 min at room temperature. Following centrifugation (2 min, 8,000 x g), samples of the supernatant (adjusted to 40–50 μg protein) were separated on a 6 % polyacrylamide gel. Immunoblot analysis was performed with an anti-CFTR antibody (NB100-92156, Novus Biologicals), followed by incubation with a secondary antibody conjugated with horseradish peroxidase. Peroxidase activity was detected by incubating with Luminata Forte Western HRP substrate (Millipore) and exposure to X-ray film (Kodak® BioMax® XAR Film).

### Salivary secretion experiments

The methods used were similar to those described by Best and Quinton [29]. In brief, mice were anaesthetized with isoflurane and maintained on a flat surface in a supine orientation with adhesive tape. The mouth was held open by cephalic retraction of the dorsal front teeth with a rubber band. An indirect heat lamp warmed the animals during the saliva collection period. Mice were pretreated with a subcutaneous injection of 50 μl (1 mM) atropine into the left cheek as a cholinergic antagonist. A pre-weighed 2 × 25 mm piece of Whatman filter paper was placed inside the injected cheek for approximately 4 min to absorb any saliva. A solution (100 μl) containing 100 μM isoprenaline and 1 mM atropine was then injected into the left cheek at the same site to induce secretion at time zero and the filter paper was replaced every three minutes for next thirty minutes. Each piece of filter paper was immediately sealed in a pre-weighed vial and the time of removal was recorded. The differential in weight of the vial pre and post secretion determined the amount of saliva secreted. The total salivary secretion was normalized to the mass of the mouse in grams. Results are expressed as μg min^−1^ g^−1^ (mean ± SEM).

## Results

### 5’-AMP-induced whole body cooling increases ΔF508-CFTR levels in CF mouse intestine

**Fig. 1:**
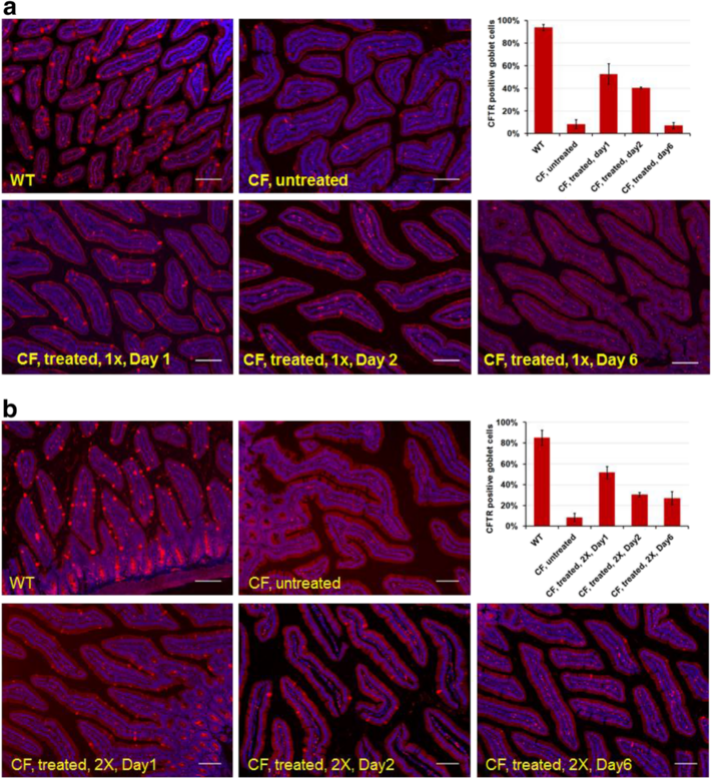
5’-AMP induced whole body cooling increases ΔF508-CFTR expression levels in CF mouse intestine. Representative cross-sections of mouse duodenal crypts from WT, untreated CF and treated CF mice were immunostained with anti-CFTR rabbit polyclonal antibody (red) and counter stained with DAPI (blue): **a** Treated CF mice that have undergone one treatment of 5’-AMP whole body cooling. **b** Treated CF mice that have undergone two treatment cycles. CF mice were sacrificed post-treatment at 24 h, 48 h and six days for tissue analysis. Quantification of ΔF508-CFTR expression was based on either positive or negative signals in intestinal goblet cells in equivalent visual fields in the respective genotype and treatment group

To investigate the effect of whole body cooling at the tissue and cellular levels, we examined CFTR expression in intestinal duodenum epithelia. Previous studies showed that CFTR is expressed mainly in the goblet cells in epithelia of intestinal duodenum in the mouse and human [30, 31]. We initiated whole body cooling of mice at a T_a_ of 15 °C by intraperitoneal (IP) injection of 5’-AMP. The T_b_ of the mice dropped to 16–17 °C within 1 h and remained at this temperature for 7–10 h before they spontaneously aroused from this deep hypometabolic state [20]. Initially, we carried out immnunostaining of intestinal epithelia from wild type and untreated CF mice. Immunostaining of CFTR was strong in the goblet cells of duodenal epithelia from wild type and was largely absent in CF mice (Fig. 1a). To assess the stability of ΔF508-CFTR protein after whole body cooling, we examined duodenal tissues from CF mice collected at 24 h, 48 h and six days post-treatment, using corresponding tissues from wild type mice as controls (Fig. 1a). We quantified the number of CFTR-positive goblet cells in selected visual fields. About 60 % of the goblet cells were positive for CFTR in treated CF mouse duodenum at 24 h post cooling. However, fewer CFTR positive cells that were expressing high levels of CFTR were apparent at 48 h post cooling. Six days after the treatment, the percentage of the CFTR positive cells in duodena from treated animals was indistinguishable from that of untreated CF mice. These findings suggest that the increase in ΔF508-CFTR stability after whole body cooling is temporary. Next, we investigated whether ΔF508-CFTR stability could be enhanced if CF mice were treated with a regime of sequential cooling. For this group, the same whole body cooling treatment was repeated 72 h after the initial cooling (Fig. 1b). At 24 h after the second treatment, immunostaining revealed that the total percentage of CFTR-positive goblet cells in CF duodenal epithelia was similar to that observed after a single cooling treatment and it declined at 48 h and six days post-treatment. However, the number of CFTR-positive goblet cells in CF mouse intestine at day six post-treatment remained significantly higher than that of untreated CF mice suggesting that repeated whole body cooling treatments have added benefit in stabilizing ΔF508-CFTR proteins.

### Increased ΔF508 CFTR stability and glycosylation following whole body cooling treatments

**Fig. 2:**
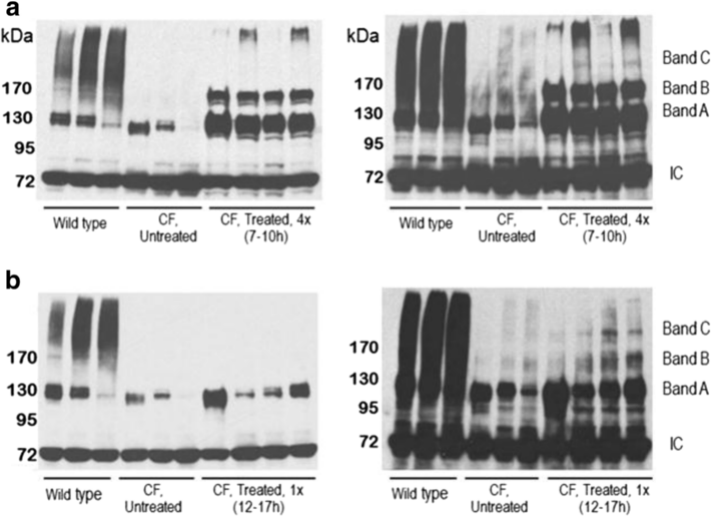
5’-AMP-induced whole body cooling treatment increases stability and glycosylation of ΔF508-CFTR in intestinal epithelium - Western analysis of intestinal duodenal epithelium. **a** CF mice given four treatment cycles every 72 h, with each cooling cycle lasting 7–10 h; **b** CF mice that had undergone one cooling treatment lasting 12–17 h. All mice were sacrificed 24 h after the last treatment. Each gel lane represents a tissue sample from an individual mouse. The left and right photograph reflects short and longer film exposures of the respective blot. Intestinal epithelia from three wild type and three untreated CF mice were used as positive and negative controls for both Western analyses. A nonspecific 72 kDa band that immune reacted with the antibody preparation was used as an internal control (IC) for loading samples

The apparent increased retention of F508-CFTR in the intestine of CF mice after whole body cooling observed by immunofluorescence staining analysis was further verified by Western analysis. Western analysis of detached intestinal duodenal epithelial cells would also reveal the glycosylation status of ΔF508-CFTR following whole body cooling treatment. Three gross glycosylation stages of CFTR have been associated with three bands observed by Western analysis: band A, the newly synthesized unglycosylated form is about 130 kDa; band B, the core-glycosylated form is about 150 kDa, and band C, the complex glycosylated form is about 170 kDa [32]. As expected, intestinal epithelial cells from individual CF mice expressed either undetectable or low levels of ΔF508-CFTR, primarily as band A. Bands B and C were generally absent in the intestinal duodenal epithelium of CF mice (Fig. 2a). In contrast, the majority of CFTR from wild type intestinal duodenal epithelia were higher molecular weight proteins migrating around 170 kDa reflecting the different glycosylated stages of the proteins corresponding to bands B to C, with a significant portion remaining in band A. The protein band pattern of wild type compared to CF intestinal epithelial cells indicated that the anti-CFTR antibodies have recognized CFTR specifically. It was noticed that a component of the polyclonal antibodies reacted with a protein band at 72 kDa, which is present in both wild type and CF epithelia. This non-specific band was used as an internal control for protein loading on the gel. Western analysis of similar tissues from CF mice that have undergone multiple cycles of whole body cooling treatments showed a marked increase in total ΔF508-CFTR retention relative to the internal control band (Fig. 2a). Compared to untreated CF mice, the level of band A and band B in the treated duodenal epithelial tissue of treated CF mice was strikingly enhanced. Upon longer exposure of the Western blot, a small amount of band C was apparent and was not detected in any of the samples from untreated CF mice. This observation raised the possibility that the cellular processing of ΔF508-CFTR was terminated prematurely possibly due to an insufficient cooling period required for the full glycosylation process.

Next, we investigated whether the level of band C was increased if we prolonged the cooling period beyond the current 7–10 h. As previously described, repeated 5’-AMP administration was not suitable to prolong the hypometabolism state, as it enhanced the mortality rate [20]. Here we used a T_a_ of 14.5 °C instead of 15 °C to prolong the hypometabolic state. Up to 17 h of cooling was achieved with this approach in 40 % of the mice. The remainder of the mice either aroused before 12 h or failed to survive the procedure. The data presented are from animals that had sustained a single cooling period of between 12 and 17 h and were sacrificed 24 h post-treatment for tissue collection. Western analysis of the duodenal epithelial tissues from four mice revealed that the relative ratio of ΔF508-CFTR in band C to internal control was increased compared to multiple cooling treatments of 7–10 h, while the relative ratio of ΔF508-CFTR in band B to internal control was decreased (Fig. 2a, b). Together, these data suggest that cooling stabilized the ΔF508-CFTR and a longer cooling period enhanced the formation of the complex glycosylated form of ΔF508-CFTR in band C *in vivo*.

### Decreased mucin accumulation in CF intestinal lumen crypts

**Fig. 3:**
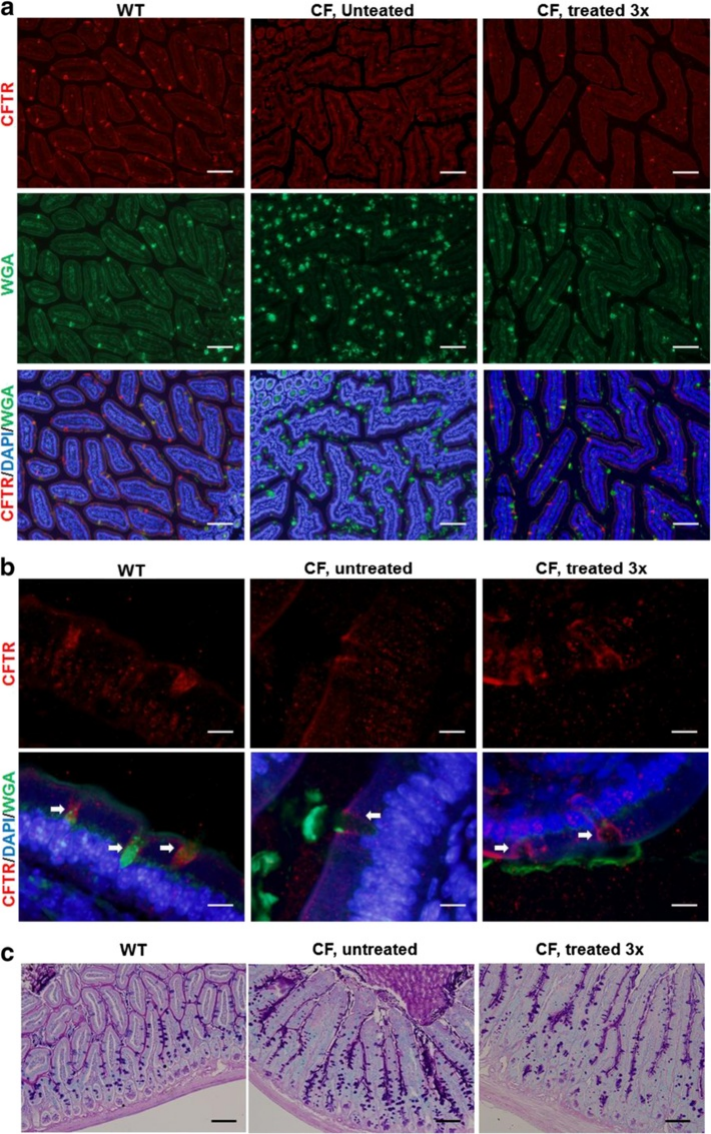
Reduced mucin levels in CF mouse duodenum crypts after whole body treatments. CFTR was immunostained with anti-CFTR antibodies (red) and mucin was stained with fluorescence labeled wheat-germ agglutinin (WGA, green). **a** Co-staining of CFTR and mucin by anti-CFTR antibodies and WGA. CF mice were either untreated or treated with three cycles of 7–10 h whole body cooling. **b** Immunostained images at 100x magnification. The arrows indicate the goblet cells. **c** PAS-Alcian-blue staining of mouse duodenum crypts from WT, CF and treated CF mice

Next we examined whether there are indicators that the enhanced levels of ΔF508-CFTR are functionally beneficial to CF mice. Mucin, the major protein component of mucus, is largely released by goblet cells in the intestine and thickened mucin in the lumen is a hallmark of CF mice. Here we subjected CF mice to three cycles of whole body cooling for 7–10 h every 72 h. Immunostaining of CFTR with the anti-CFTR antibody and co-staining of mucin with fluorescence labeled wheat germ agglutinin (WGA) were carried out using isolated intestinal crypts from wild type and treated and untreated CF mice. WGA, which binds to sialic acid and N-acetyl-D-glucosamine on the mucin protein, was used to detect the presence of mucin. DAPI counterstaining was also carried out to detect nuclei. The WGA staining in wild type was largely located within the goblet cells indicating pre-secreted mucin and mucin staining were not apparent in the lumen of intestinal crypts. By contrast, we observed prominent WGA staining in the lumen, particularly at the goblet cell outlet of untreated CF intestinal crypts, in addition to staining in goblet cells (Fig. 3a), WGA staining of treated CF samples showed that the majority of mucin remained within the goblet cells similar to that observed in wild type, while the number of strong mucin staining cells in the lumen was highly reduced compared with untreated CF mice. Further, many WGA positive goblet cells were also positively stained by anti-CFTR antibodies in the intestinal tissues from treated but not untreated CF mice (Fig. 3a, b). At a higher resolution, WGA mucin staining appeared as a weak signal in wild type lumen and much less concentrated near the goblet cell outlet of treated CF mice comparing to untreated CF mice (Fig. 3b). Using PAS staining, accumulation of mucus released from goblet cells in the crypt lumen and along the villous surfaces was very prominent in CF but absent in wild type animals (Fig. 3c). The accumulation of mucus in the intestinal crypt lumen of CF mice was visibly reduced, accompanied by a visibly reduced lumen mucus density, after repeated whole body cooling treatment. These observations suggest that after whole body cooling treatment, increased ΔF508-CFTR protein stability correlates with reduced accumulation of mucin in the intestinal lumen of CF mice.

### Increased β-adrenergic-dependent salivary secretion in treated CF mice

**Fig. 4:**
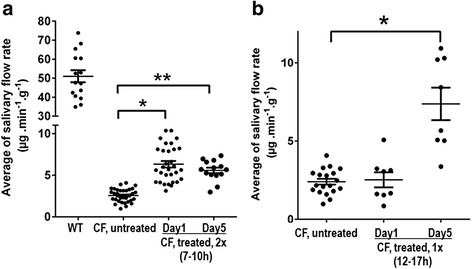
β-adrenergic dependent salivary secretion is enhanced after whole body cooling treatment. The level of β-adrenergic dependent salivary secretion following isoprenaline injection: **a** Wild type, untreated and treated CF mice (two cycles of treatment) at 24 h and 5 days post-treatment. **b** Untreated CF versus CF mice treated with a 12–17 h cooling regime at 24 h and day five post-treatment. Each circle in each group represents an individual mouse. * indicates statistical analysis SEM (*P* < 0.05)

Human sweat glands normally respond to both β-adrenergic and cholinergic stimulation [33], but in CF patients these glands fail to respond to stimulation with isoprenaline, a β-adrenergic agonist [34]. Mice do not have thermoregulatory sweat glands like humans, however the salivary gland in mouse responds in the same way as human sweat glands to β-adrenergic and cholinergic stimulation. The restoration of this β-adrenergic-dependent activity has been previously used as a functional assay to evaluate the effectiveness of potential drug therapy in restoration of CFTR function *in vivo* [29]. We compared average salivary flow rates in response to the β-adrenergic agonist isoprenaline in wild type controls and in AIHM whole body treated and untreated CF mice. After blocking cholinergic dependent secretion with atropine, isoprenaline induced an increased salivary secretion with an average flow rate of 51.04 ± 3.16 μg min^−1^ g^−1^ in wild type mice. Consistent with previous observations, untreated CF mice (*n* = 30) showed a very weak salivary secretion response to isoprenaline stimulation, with an average flow rate of 2.56 ± 0.15 μg min^−1^ g^−1^. The isoprenaline-dependent salivary secretion improved significantly in treated CF mice (*n* = 31), with an average rate of 6.32 ± 0.40 μg min^−1^ g^−1^ 1 day after two cycles of whole body cooling treatments. Unlike untreated CF mice, where the salivary flow rate measurements formed a tight cluster around 2.56 μg min^−1^ g^−1^, the flow rate pattern distribution from individual treated CF mice ranged up to more than 10 μg min^−1^ g^−1^. Five days post cooling treatment, salivary secretion dropped to 5.58 ± 0.33 μg min^−1^ g^−1^ but remained higher than that of untreated CF mice (Fig. 4a).

Next we investigated whether a single treatment of prolonged cooling (12–17 h) would result in greater improvement than the multiple cooling regimes. Surprisingly, salivary secretion didn’t noticeably improve in CF mice one day post prolonged cooling treatment. However, salivary secretion significantly improved 5 days post prolonged cooling treatment, with an average rate of 7.38 ± 1.03 μg min^−1^ g^−1^ (Fig. 4b). We reason that the apparent absence of improvement at day one after prolonged cooling treatment could have been due to the lack of fluid intake in treated mice following the prolonged stupor state. Together, these findings suggest improved function of the stabilized ΔF508-CFTR after whole body cooling treatment *in vivo*.

### 5’-AMP induced whole body cooling treatments improve CF mouse survival

**Fig. 5:**
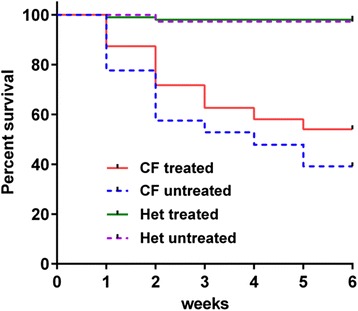
Logrank analyses of survival rates. The graph represents the Logrank analyses of survival rates of groups of treated and untreated CF mice and heterozygote (Het) littermates; The Logrank tests (low panels) show the statistical differences among survival curves. It shows that the cooling treatment used in this study didn’t impact the survival of the heterozygote CF mice, while improved the survival of CF mice significantly. Thus, the treatment does not appear to impact the animals negatively

Finally, we examined whether CF mice that were given multiple treatments with the AIHM whole body cooling regime had reduced mortality. We reasoned that improved mutant protein processing and trafficking and at least partially restored CFTR function *in vivo*, could result in an improvement in the survival rate of the CF mice. Previous studies have shown that ΔF508-CFTR mice have a very high mortality rate up to 6 weeks of age, with less than 40 % survival compared with over 95 % for wild type littermates [24]. The mortality rate of CF mice older than six weeks of age dropped significantly. To generate a large number of CF animals for this study, we bred a homozygote CF male with heterozygote CF female animals. Mice that are homozygous for ΔF508-CFTR are significantly smaller than their heterozygous siblings, a characteristic apparent by one week of age. Genotyping a cohort of smaller versus normal size sibling revealed a tight correlation between small pup sizes and homozygotes for ΔF508-CFTR. Therefore, we used size differential at age seven days as an initial screen to identify mice homozygous for ΔF508-CFTR. To investigate whether whole body cooling improves survival of CF mice, starting at age seven days, CF mice were treated with whole body cooling twice a week for two weeks. To ascertain possible small improvements, a large cohort of treated mice versus untreated controls was examined. At the completion of the studies, genomic DNA samples were collected from all the mice and genotyped for homozygosity of the ΔF508 mutation. As shown in Fig. 5, the overall mortality of CF mice was significantly decreased in the treated group compared to the untreated group. By the age of five weeks, the cohorts that underwent whole body cooling treatment displayed an improvement in survival rate to 54 % (*n* = 198), compared with 39 % (*n* = 278) in untreated CF mice. Noticeably, the biggest improvement in survival was between weeks one and two of age following the onset of treatment. The differential mortality rate between treated and untreated CF mice remained apparent at the end of the experiment at six weeks of age. Together, these findings showed that whole body cooling has a quantifiable beneficial impact on the overall survival of CF mice.

## Discussion

Until recently, mammals in deep hypometabolism with severe T_b_ reduction and stupor were only observed in nature during hibernation and torpor [35]. Lowering body temperature in mammals artificially has not only been considered difficult but also dangerous, as it can induce cardiac arrest. Our studies were the first to demonstrate that the metabolite 5’-AMP can induce mice to undergo transient hypometabolism with a T_b_ as low as 26 °C when maintained at room temperature [21]. Since then, our studies have shown that mice given 5’-AMP can have their T_b_ safely reduced to 16–17 °C when kept at a T_a_ of 15 °C and remain in this deep hypometabolic state for 7–10 h [20, 22, 23, 36]. AIHM is now recognized as an inducible form of torpor by investigators who have studied natural torpor [37]. Recently, a number of independent studies have used AIHM to reduce body temperature in order to investigate animal models of clinical disorders thought to benefit by severe T_b_ reduction [38–40]. These studies demonstrate that AIHM whole body cooling is safe when used under the appropriate conditions and could provide a beneficial treatment for these disorders.

Here, we investigated whether whole body cooling could rescue misfolded proteins *in vivo*. One of the best characterized temperature-sensitive misfolded proteins is ΔF508-CFTR, the most common mutation responsible for CF in humans. Animal models of cystic fibrosis have provided a powerful tool for studies of the mechanisms and complexities of the human CF disease. Understandably, the murine ΔF508-CFTR model has inherent limitations due to anatomic and immunologic differences between mice and humans. The ΔF508-CFTR CF mice do not develop the lung inflammatory disorders caused by chronic bacterial infections observed in human CF patients [24, 41]. One of the difficulties in dealing with this CF mouse model is the narrow window, the first month post-natal, within which the CF pathology has the most severe impact on the animals. Whole body cooling of mice seven days post-natal had not been tried before. Our studies show that the AIHM mediated cooling treatment appeared well tolerated by mice even at this age. A hallmark of ΔF508-CFTR CF mouse models is the severe intestinal pathology, including goblet cell hyperplasia, mucin accumulation in the crypts of Lieberkuhn, crypt dilation and intestinal obstruction [42]. These patho-physiological features mirror the meconium ileus observed in human infants with CF [26]. Thus, we chose to examine intestinal duodenum tissue to evaluate the effect on ΔF508-CFTR stabilization by AIHM whole body cooling *in vivo*. After whole body cooling treatment, there was an increase in ΔF508-CFTR stability in duodenum goblet cells. We reasoned that this increase could be due to stabilization of misfolded mutant ΔF508-CFTR proteins, similar to that observed after low temperature treatment of ΔF508-CFTR expressing cultured cells [16, 17]. The improvement in retention of the mutant CFTR proteins was further verified by Western analysis. However, Western analysis revealed that whole body cooling treatment fell short in rescuing ΔF508-CFTR fully. Although the protein levels were highly elevated after the cooling treatments, the majority of ΔF508-CFTR retained was either in band A (unglycosylated) or band B (partially glycosylated), with only trace levels of the fully glycosylated form present in band C. Multiple short cooling treatments only enhanced bands A and B suggesting that the processing of misfolded protein to band C may be a continuous event. Consistent with this reasoning, application of a longer period of cooling enhanced the fraction of fully rescued ΔF508-CFTR in band C. Our findings are consistent with those from cell culture studies, which showed that a cooling period of at least 16 h is necessary for full glycosylation of ΔF508-CFTR [17]. Although the current observed effect was limited by our present cooling technology, our findings support the possibility that full glycosylation of ΔF508-CFTR levels will be enhanced by longer cooling periods *in vivo*.

Previous studies with cultured cells have reported that CFTR protein in band B has a similar electrophysiological function to that of band C [32]. The most effective way to gauge ΔF508-CFTR rescue at the organismal level is to measure the correction or attenuation of ΔF508-CFTR associated pathological phenotypes in response to treatment. First, we examined whether the whole body cooling treatment significantly reduced mucin accumulation in the crypts of Lieberkuhn, a key hallmark phenotype in CF mouse intestine. After PAS staining, the reduction in mucin accumulation was visibly apparent in treated compared to untreated CF mice. WGA staining confirmed the prominent mucin accumulation around the goblet cells in untreated CF intestinal crypt lumen and its significant reduction in treated CF mice. In contrast, wild type intestinal crypts displayed pre-secreted mucin within the goblet cells and no significant staining of accumulated mucins in the lumen was apparent.

The mucin accumulation observations are descriptive assessments and not quantitative measurements of function. Therefore, we measured β-adrenergic dependent salivary secretion in CF mice to provide quantitative evidence of functional rescue *in vivo*. This analysis of salivary gland function also extended our study to another organ severely affected by ΔF508-CFTR in mice. Consistent with previous findings, β-adrenergic dependent salivary secretions in CF animals were severely dampened compared with wild type mice [29, 43]. However, after whole body cooling, a moderate but significant improvement in β-adrenergic dependent salivary secretion was apparent in treated compared with untreated CF mice. While the average β-adrenergic dependent salivary secretion in treated CF mice was far from the levels observed in wild type animals, the upper secretion levels observed in some treated CF mice approached 15–20 % of the average secretion value observed in wild type mice. Significantly, none of the untreated CF mice in the large cohorts displayed β-adrenergic dependent salivary secretion of similar magnitude. We reason that the variable individual responses could result from the unevenness in the length of cooling for each mouse. The variability in the length of AIHM results from the variability in the onset of arousal from a torpor-like state by individual animals. At present, our ability to control the onset of arousal in mice under AIHM remains limited. In the study using prolonged cooling, there was no apparent early improvement in β-adrenergic dependent salivary secretion at 24 h post-treatment. This is likely due to the fact that these mice may not have fully recovered from 12 to 17 h in a state of deep stupor. When fully recovered, measurements at five days post-treatment revealed that these cohorts of treated mice on average showed moderately better β-adrenergic dependent salivary secretion than those that had undergone multiple regimes of shorter whole body cooling treatments.

Finally, we examined the apparent efficacy of cooling based on post weaning survival of the CF mice. Previous studies have shown that mortality of this genetically modified line of CF pups is very high especially during the period prior to weaning. We observed a 15 % improvement in survival in treated versus untreated CF pups. This differential impact on survival was especially evident in the first week of treatment. After treatment stopped, the differential in mortality between treated and untreated CF mice narrowed slightly but was mostly maintained.

Many other human diseases have been associated with temperature-sensitive protein misfolding defects. Low temperature treatments had been shown to be effective in correcting such defects in cell culture. Our goal in this study was to use the ΔF508-CFTR mouse model to investigate whether reduction in body temperature of the animal can recapitulate the cell culture observations by rescuing temperature-sensitive misfolded proteins thus enhancing their stability and function. Interestingly, a recent study demonstrated that AMP-induced whole body cooling in both prion infected and Alzheimer-type mouse models could activate a cold stress response that promotes synapse regeneration, suggesting a novel therapeutic approach for neurodegenerative disorders [44].

## Conclusions

Our investigation demonstrates that increased stabilization of ΔF508-CFTR *in vivo* can be achieved by whole body cooling. The increased levels of ΔF508-CFTR confer improvement in CFTR functions, alleviate CF pathological phenotypes and decrease mortality in CF mice. These findings open up the possibility that further advances in whole body cooling techniques may offer treatment of different disorders arising from temperature-sensitive misfolding defects.

## Abbreviations

5’-AMP: 5’-Adenosine monophosphate
AIHM: 5’-AMP induced hypometabolism
CF: Cystic Fibrosis
CFTR: Cystic fibrosis transmembrane conductance regulator
DAPI: 4′,6-diamidino-2-phenylindole
ER: Endoplasmic reticulum
*in vitro*: Latin: in glass; for the effects out of tests on cells or biological molecules testing dishes
*in vivo*: Latin for “within the living”; for the effects out of tests on whole, living organisms
IP: Intraperitoneal injection
kDa: Kilodalton
PAS: Periodic acid–Schiff, used in a staining method to detect polysaccharides in mucins
T_a_: Ambient temperature
T_b_: Core body temperature
WGA: Wheat germ agglutinin
WT: Wild type
ΔF508: A deletion of a phenylalanine at amino acid position 508 of the CFTR protein
